# The effect of a gratitude program based on positive thinking on nurses' resilience in the post-Coronavirus 2019 pandemic era

**DOI:** 10.17533/udea.iee.v43n1e07

**Published:** 2025-04-28

**Authors:** Ali Dehghani

**Affiliations:** 1 Associate professor, Department of Community Health Nursing, School of Nursing, Jahrom University of Medical Sciences, Jahrom, Iran. Email: ali.dehghani2000@gmail.com. Corresponding Author. https://orcid.org/0000-0002-1768-1856 Jahrom University of Medical Sciences Department of Community Health Nursing School of Nursing Jahrom University of Medical Sciences Jahrom Iran ali.dehghani2000@gmail.com

**Keywords:** positive thinking, gratitude, resilience, nurse, Post-COVID condition., optimismo, gratitud, resiliencia, enfermeras, condición post-COVID, otimismo, gratidão, resiliência, enfermeiros, condição pós-COVID

## Abstract

**Objective.:**

To evaluate the effect of a gratitude program based on positive thinking on nurses' resilience in the post-Coronavirus pandemic era**.**

**Methods.:**

This is a quasi-experimental study. Eighty nurses in Peimanieh Hospital affiliated to Jahrom University of Medical Sciences were selected using convenience sampling method. They were randomly assigned to the intervention group (*n*=40) and control groups (*n*=40) from September to December 2023 in the southern of Iran. The intervention group were given a gratitude plan based on positive thinking in 28 days using WhatsApp in the form of daily. Training content were according to the positive psychotherapy and gratitude of Seligman and Rhonda Byrne. The subjects of control group were not taught. Data were gathered using Connor-Davidson Resilience Scale.

**Results.:**

The findings determined that immediately and two months after the intervention the total mean resilience scores had significant differences between the two groups (*p*<0.001). Repeated measures testing revealed significant improvements in total mean resilience scores from baseline to two months post-intervention (*p*=0.002), while between time points in the control group was not significant difference (*p=*0.32).

**Conclusion.:**

The findings provide evidence for the use of a gratitude programme, based on positive thinking, to increase resilience in nurses in the post-Coronavirus 2019 era.

## Resumen

**Objetivo.:**

Evaluar el efecto de un programa de gratitud basado en el pensamiento positivo sobre la resiliencia de las enfermeras en la era posterior a la pandemia del coronavirus.

**Métodos.:**

Se trata de un estudio cuasi-experimental en el que se seleccionaron por muestreo por conveniencia a 80 enfermeras del Hospital Peimanieh, afiliado a la Universidad de Ciencias Médicas Jahrom (Irán), las cuales fueron asignadas aleatoriamente a los grupos de intervención (*n*=40) y de control (*n*=40). La recolección de la información se realizó entre septiembre y diciembre de 2023. El grupo de intervención recibió un programa educativo de gratitud fundamentado en el pensamiento positivo durante 28 días utilizando WhatsApp. El contenido de la intervención se basó en la psicoterapia positiva de Seligman y en el poder de la gratitud y Rhonda Byrne. Los sujetos del grupo de control no recibieron formación. Los datos se recogieron utilizando la Escala de Resiliencia de Connor-Davidson.

**Resultados.:**

Los hallazgos mostraron que, inmediatamente y dos meses después de terminada la intervención, las puntuaciones medias totales de resiliencia presentaban diferencias significativas entre los dos grupos (*p*<0.001). Las pruebas de medidas repetidas revelaron mejoras significativas en las puntuaciones medias totales de resiliencia desde el inicio hasta dos meses después de finalizada la intervención (*p*=0.002), mientras que entre los puntos temporales en el grupo de control no hubo diferencias significativas (*p*=0.32).

**Conclusión.:**

Los resultados proporcionan evidencia para el uso de un programa de gratitud basado en el pensamiento positivo, para aumentar la resiliencia en las enfermeras en la era post-Coronavirus 2019.

## Resumo

**Objetivo.:**

Avaliar o efeito de um programa de gratidão baseado no pensamento positivo na resiliência dos enfermeiros na era pós-pandemia do coronavírus.

**Métodos.:**

Este é um estudo quase experimental no qual 80 enfermeiros do Hospital Peimanieh, afiliado à Universidade de Ciências Médicas Jahrom (Irã), foram selecionados por amostragem de conveniência e designados aleatoriamente para grupos de intervenção (*n*=40) e controle (*n*=40). A coleta de dados foi realizada entre setembro e dezembro de 2023. O grupo de intervenção recebeu um programa de educação sobre gratidão baseado em pensamento positivo por 28 dias usando o WhatsApp. O conteúdo da intervenção foi baseado na psicoterapia positiva de Seligman e no poder da gratidão e em Rhonda Byrne. Os indivíduos do grupo de controle não receberam treinamento. Os dados foram coletados usando a Escala de Resiliência de Connor-Davidson.

**Resultados.:**

Os resultados mostraram que imediatamente e dois meses após o término da intervenção, as pontuações médias de resiliência total apresentaram diferenças significativas entre os dois grupos (*p*<0.001). Testes de medidas repetidas revelaram melhorias significativas nas pontuações médias de resiliência total desde o início até dois meses após o término da intervenção (*p*=0.002), enquanto entre os pontos de tempo no grupo de controle não houve diferenças significativas (*p*=0.32).

**Conclusão.:**

Os resultados fornecem evidências para o uso de um programa de gratidão baseado no pensamento positivo, para aumentar a resiliência em enfermeiros na era pós-Coronavírus 2019.

## Introduction

In 2020, the World Health Organization classified the coronavirus infection as a pandemic. This virus has caused many pressure on healthcare systems everywhere the universe. Injuries to healthcare workers are on the rise due to rising infections, and lack of access to necessary personal protective equipment and medical beds. Nursing staff are in the early stages of the health crisis and are facing many challenges caused by COVID-19.^1^ Lorente *et al*. found that in addition to COVID-19, the possibility of infection, heavy workload, and inadequate preparation were related with mental health impairments among caregivers, but these impairments could be reversed. The author said it was controlled by resilience.^2^ Another study found that upper resilience and positivity were related with less burnout, negative emotions, and emotional exhaustion.^3^

One of the variables that plays an important role in the nurse's profession is resilience.The resilience as an approach that allows individual to compatibility to undesirable and keep hope.^4^ Resilience criteria assess an individual's ability to seek out and utilize support networks, increase self-awareness and accept situations, and grow after stressful situations.^5^ Positive thinking is a new event in the psychological society that relies on need to understand the positive aspects of human experience and what makes life worth living.^6^ Previous research has shown the influence of positive thinking on mental health indexes in people with chronic illnesses.^7^^,^^8^ One aspect of positive thinking is gratitude, which makes people happier, more hopeful, and more satisfied with life.^9^ Gratitude interventions lead to significant improvements in psychological, spiritual, and physical health. Because grateful people are stronger than others at forming social engagements, using stress management skills, being inclusive, and working creatively to solve problems.^10^

The use of social media as a training approach has been introduced to make conveying concepts and materials easier, more comprehensive, and more engaging through text, audio, images, and video, and this method is now widely used for concept conveyance.^11^ The unique features of PC- and phone-based multimedia training programs allow you to overcome the limitations of traditional training, including how to understand the current situation of the new coronavirus infection outbreak. Therefore, this study aims to examine the effects of a gratitude program based on positive thinking using multimedia on nurses' resilience, taking into consideration the mental health of the nursing profession and nurses in the post-coronavirus pandemic era. 

## Methods

This study is a quasi-experimental study conducted at the Peimanieh Hospital affiliated to Jahrom University of Medical Sciences from September to December 2023, Iran. Participation criteria, absence of psychological problems, and intention to participate in the study of nursing staff in the new coronavirus infection ward. Exclusion criteria were not completing the questionnaire or attending the training course. In this study, 80 nurses were selected using convenience sampling method from among nurses who had the study inclusion criteria and after randomly divided into control and intervention groups. Due to the limited number of nurses who had the study inclusion criteria and were willing to participate in the study, random sampling was not possible and randomization was done only in the distribution of samples into intervention and control groups. The intervention type was assigned to nurses randomly using permuted block randomization with a block size of 10 (using the table on random permutation). Blinding was not performed.

Data were collected through demographic information and the scale Connor-Davidson Resilience Scale (CD-RISC). The CD-RISC included 25 questions distributed in 5 dimensions, each item is rated using a five-point (1=Strongly disagree, 2=Disagree, 3=Neutral, 4=Agree and 5=Strongly agree). The score for each dimension was determined by summing the scores for that question. The overall resilience score is the sum of the scores for all questions range from zero to 100, with higher values ​​indicating more resilience.^12^ The validity of scale was confirmed by Mohammadi, with Cronbach’s alpha being 0.89^13^ In the current research, this coefficient obtained was 0.77.

In the intervention group, training and practice on gratitude program based on positive thinking through multimedia application (WhatsApp) was conducted in 28 days in the form of daily. Training content were according to the positive psychotherapy and gratitude of Seligman and Rhonda Byrne.^14^^,^^15^ Contents discussed in every session with more details in the appendix data. Instructional methods include video clips and audio files that correspond to the training topics.

Ethical considerations. This study was confirmed by the ethics committee of Jahrom University of Medical Sciences (Ethics Number IR.JUMS.REC.1400.051). All the participants signed an informed consent. The aims and approach of the research were explained to them and they were then given adequate trust regarding the confidentiality of the data. In addition to, after the completion of the study and for meet the study ethics, the educational contents for the control group were accomplished.

Data analysis. Information was analyzed using SPSS V.21. Shapiro-Wilk tests of data distribution, chi-square tests, independent t tests, repeated measures, and LSD post hoc tests were used. The significance level was assumed to be *p*<0.05.

## Results

There were five persons withdrew in the control group. One person did not participate in the educational sessions in the intervention group; in the end, 39 nurses participate in the intervention group and 35 persons participate in the control group.

Hence, six of the participants were withdrawn from the study during the study due to lack of follow-up. Based on the findings, the demographic characteristics were the same in both groups (*p*>0.05) (Table 1). 

**Table 1:** Frequency distribution of demographic variables in the study groups

Variable	Group			***p*-value**
	**Intervention (*n*=39)**	**Control (*n*=35)**	
**Gender; *n* (%)**			
Male	5 (12.8)	5 (14.3)	0.52^*^
Female	34 (87.2)	30 (85.7)	
**Marital status; *n* (%)**			
Single	10 (25.6)	7 (20)	0.29^*^
Married	29 (74.4)	28 (80)	
**Educational status; *n* (%)**			
Bachelor of Nursing	35 (89.8)	31 (88.6)	0.70^*^
Master of Nursing	4 (10.3)	4 (11.4)	
Age; (Mean±SD)	37.05 ± 5.67	35.23 ± 8.38	0.27^**^
Work experience; (Mean±SD)	3.33 ± 1.10	3.34 ± 1.71	0.97^**^

* Chi-square, ** Independent samples test

**Diagram 1:**
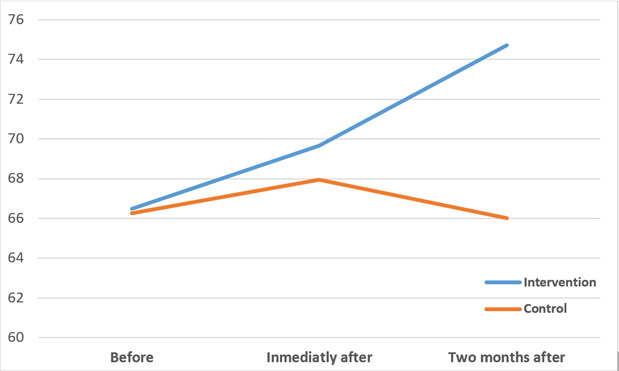
Total mean scores of resilience before, immediately, and two months after the intervention in the two groups

The between the two groups regarding the resilience score and its dimensions no significant differences were observed in the before the intervention (*p*>0.05). Immediately after the intervention, a significant difference in resilience scores was observed between the two groups (*p<*0.05) for the total and for three of the five dimensions (positive acceptance of change and secure relationships, control and spiritual influences). Furthermore, between the two groups in the 2 months after the intervention was observed significant differences in the total and dimensions scores of the CD-RISC, although the mean value was upper in the intervention group (*p*<0.001). Repeated measurements showed that mean resilience improved significantly over time in the intervention group (*p*<0.001) (Table 2) (Diagram 1).

**Table 2:** Comparison of the mean total scores of resilience and its subscales before, immediately, and two months after the intervention in the two groups

Variable	Group	Time			***p*-value**
		Before intervention	Immediately after intervention	Two months after intervention	
Total resilience	Intervention	66.48 ± 17.78	69.66 ± 4.74	74.71 ± 7.44	0.002
Control	66.25 ± 9.03	67.94 ± 10.50	66.02 ± 8.41	0.32
*p*-value	0.94	0.03	0.0001	
Personal competence	Intervention	18.94 ± 5.78	19.97 ± 2.37	21.74 ± 2.80	0.0001
	Control	19.42 ± 3.96	19.96 ± 2.40	19.82 ± 2.58	0.16
	*p*-value	0.12	0.18	0.001	
Trust in one’s instincts and tolerance of negative Affect	Intervention	17.66 ± 0.70	18.02 ± 0.54	23.67 ± 0.21	0.001
	Control	17.91 ± 0.74	18.82 ± 0.57	18.88 ± 0.52	0.63
	*p*-value	0.32	0.13	0.0001	
Positive acceptance of change and secure relationships	Intervention	13.33 ± 3.99	15.48 ± 2.19	16.41 ± 2.14	0.016
	Control	13.17 ± 3.11	13.62 ± 3.91	12.71 ± 3.65	0.12
	*p*-value	0.84	0.013	0.0001	
Control	Intervention	8.58 ± 2.90	10.02 ± 1.87	13.65 ± 1.22	0.02
	Control	7.45 ± 2.89	7.00 ± 2.87	8.05 ± 2.33	0.18
	*p*-value	0.09	0.012	0.013	
Spiritual influences	Intervention	5.07 ± 2.45	6.98 ± 1.61	8.24 ± 1.14	0.01
	Control	5.57 ± 1.98	5.59 ± 2.01	5.32 ± 1.95	0.30
*p*-value	0.34	0.04	0.0001	

## Discussion

The findings of this research showed that in the intervention group, the mean resilience of the nurses immediately and two months after the intervention improved compared to before the intervention; however, a significant difference was not observed in the resilience of the control group. This findings revealed significant improvements in mean resilience from baseline to two months after the intervention, while between time points in the control group was not significant difference. Research has shown that positive thinking gratitude programs promote resilience in caregivers. The results suggest that such programs can help improve nurses' resilience and its dimensions over time, especially in the post-COVID-19 pandemic. Matel-Anderson *et al.*^16^ performed a cross-sectional and correlational study on 131 college students those results suggested an impact of self-esteem on resilience using positive thinking. Results from Mehafarid *et al*.^17^ This study found that positive thinking education influenced nurses' resilience, resiliency, and burnout. Also, the studies showed optimism training and positive thinking in the form of past life events and modifying them by setting positive goals for the future, has been effective in increasing self-efficacy, meaning and hope for life and well-being of individuals.^18^ In the studies such as Seligman *et al.*,^19^ and Luthans *et al*.^20^ the impact of positive thinking education psychological intervention plan in improving work quality and resilience in hard working conditions as well as the positive effect it has on burnout has been considered. This finding that the effectiveness of positive thinking and optimism teaching techniques was consistent with present research including McCalister *et al*.^21^ and Friborg *et al*.^22^ The findings of the present study and related studies show that using gratitude programs based on positive thinking over a longer period of time can have better effects on improving the resilience and psychological state of the participants. Also, the use of gratitude interventions over time leads to a significant increase in individuals' mental, emotional, and physical well-being, because grateful people are more capable than others in terms of forming social commitments, using stress coping skills, and problem-solving.^10^ Therefore, in the present study, the difference in resilience scores was greater two months after the end of the intervention.

Explaining this finding, it can be said that positive thinking and optimism training programs aimed at promoting resilience and acceptance or coping with the realities of the coronavirus and its consequences for nurses could increase the resilience rate of nurses. The conclusion of this finding is that people with high resilience, in stressful situations and unfortunate situations such as post-COVID - 19 pandemic, maintain their psychological health and have psychological adaptation, thereby increasing their performance and feeling of satisfaction.^23^ Other studies have shown that nurses who have strong positive thoughts feel better. Research has confirmed the effectiveness of positive thinking in improving health tolerance, physical activity, social functioning, and quality of life.^24^ Bagheri Charook *et al*.^25^ confirm that teaching positive thinking strategies leads to improved functioning and productivity. Hence, in explaining the above results, it can be said that it is significant to pay attention to the characteristics and positive aspects of behaviors instead of focusing on the weaknesses and negative aspects of behavior. 

The study findings highlighted that positive thinking programs lead to the promotion of resilience in nurses. Enhancing resilience in nursing, especially during times of crisis and after that, has an effective role in increasing the spirit of nursing as well as the quality of nursing care. This study emphasizes that the use of social media education in the post-crisis era allows nurses to engage with positive thinking content and improve their resilience in order to enhance their knowledge and practice. Positive thinking programs may help with motivation and retention among health professionals. Gratitude programme based on positive thinking is associated with a healthy work environment among nurses.

From the limitations of the current research is that the samples were chosen trough census, therefore the future researches would rather to be executed on a more nurses and trough random sampling. Another limitation of this study is that only one instrument was used to assess the resilience.

Conclusion. The findings provide evidence for the use of a gratitude programme based on positive thinking, to increase resilience in nurses in the post-Coronavirus 2019 era. This study also shows the importance and potential of social media programs in positive thinking and resilience educational curricula. 

## Appendix

The gratitude training program based on positive thinking in 28 days in the intervention group

Day 1: Count Your Blessings: “better to count blessings than to lose blessings to counting your troubles”

Day 2: Magic Rock: Find a rock/stone-small to fit in the palm, smooth. Put where you can see it -near bed.

Day 3: Magical relationships: People with gratitude have better relationships in family, with friends, in office, outside.

Day 4: magical Health. Thank you for everything in the body-list

Day 5: Magic Money If you have lack of money, worry, envy, jealousy, disappointment, discouragement, doubts, fear, keep it more away.

Day 6 : Works like magic " If you take any activity, any skill-take it & push it as far it goes & push it beyond where it has never been before, push it beyond the edges of edges, then you force it in the realm of magic". Tom Robbins.

Day 7: Magical Way out of Negativity a thankful person is thankful under any circumstances. It is impossible to be /critical/blame/sad, when you are grateful. First, as difficult as it may be, you have to look for things to be grateful for in a situation. No matter how bad things are, you can always find something to be grateful for.

Day 8: Magic Ingredient be grateful for food and drink, every time you eat or drink something.

Day 9: Money Magnet Complaint makes you poorer, gratitude-richer. True for money/happiness.

Day 10: Magic Dust Everyone Ancient spiritual teaching: What we give to others with full heart returns multiplied many fold.

Day 11: Magic Morning When you arise in the morning, think of what a privilege it is to be alive, to think, to enjoy, to love.

Day 12: Magical People Who Made a Difference “At times, our own light goes out and is rekindled by a spark from another person". Each one of us has a cause to think with deep gratitude of those who have lighted the flame within us. Albert Schweitzer (Nobel Peace prize winner).

Day 13: make All Your Wishes Come True Gratitude is a must both before and after you receive something. Normally we do it only after receiving.

Day 14: Have a magical Day In the morning, before getting out of bed, or shaving, shower, list plans for the day & say ‘Thank You’ for each one going well. Wipes out unexpected problems and difficulties.

Day 15: Magically Heal Your Relationships when we have difficult relationship, these can improve ONLY when we are grateful to the other person at least for a few things. Blame, anger or hatred makes relationships worse. These burn your life. Gratitude heals.

Day 16: Magic and Miracles in Health Gratitude’s magical power increases the natural flow of health to the mind and body & assists quick healing. Helps other body care: Exercise, medicines, good food.

Day 17: The magic Check When you direct gratitude’s magical power toward any condition, a new condition is created, eliminating the old.

Day 18: The Magical To-Do List Every day we seem to have so many things to do that we feel overwhelmed. Applies to everyone, even a house wife. When we don't know what to do, where to start, we feel depressed, and tend to give up.

Day 19 : Magical Footsteps-Very Good Mood Lifter A hundred times every day I remind myself that my inner and outer life depend on labors of other men, living/dead, & that I must exert myself in order to give back in some measure, as I have received, and am still receiving.

Day 20: Heart Magic Focus your mind on the area of heart. Close your eyes as you say, ' Thank You'. Gratitude leads to harmony resulting in improved immune system and health.

Day 21: Magnificent Outcomes Say grace before any important activity you want to be successful, interview, exam, lecture, meeting, purchase.

Day 22: Before Your Very Eyes Practice: 1. Count your blessings 2. Start of the day: Take your top 10 desire list. Read each desire & for one minute imagine & visualize that desire to be fulfilled. Feel gratitude. 3. Carry desire list. On two occasions; read and feel gratitude 4. Magic Rock.

Day 23: The Magical Air That You Breathe Practice: 1. Count Blessings 2. Five times today, stop and think about the glorious air that you breathe. Take five deliberate breaths, and feel the air going in/out and joy. 3. Magic Rock.

Day 24: The Magic Wand- IMPORTANT-But see if it can be made better People who wait for a magic wand fail to see that THEY ARE THE MAGIC WAND. Thomas Leonard (Life Coach).

Day 25: Cue the magic: Very Good Use people, circumstances, events that surround you daily to provide clues to be grateful for. E.g. Ambulance -Thank you for good health, Police- Thank You for safety.

Day 26: Magically Transform Mistakes into Blessings- Very Good Turn wounds into wisdom Oprah Winfrey.

Day 27: Magic Mirror- Very Good “The appearance of things changes according to the emotions, and thus we see magic and beauty in them, while the magic and beauty are really in ourselves" Kahlil Gibran.

Day 28: Remember The Magic “That’s the thing with magic you have got to know/feel. It is still here, all around us, or it just stays invisible for you". Charles de Lint In the morning, remember yesterday's blessings. Make a list, say aloud. If you can't find many for the previous day, find from previous days.

Also, Positive thinking training programs including the following according to Seligman were used in the training sessions:

1. Explanation of procedures and reasons for our specific selections, introduction to the concept of positive thinking, group introductions and a review of rules

2. Factors affecting health, familiarity with changeable and unchangeable elements in life

3. Steps to accepting the unchangeable conditions of life, ways to deal with unchangeable conditions in life

4. Ways to overcome depression, specification of values and goals in life

5. Assessment of satisfaction with life and the ability to live happily, being positive by challenging negative thoughts, use of productive language and reconsideration in beliefs

6. Anger Management

7. Connecting with the present time through mindfulness meditation

8. Experiencing the present time through mindfulness and a recapitulation of contents presented during the course
